# Predicting postmortem neuropathology of Alzheimer’s disease and related dementias using widely accessible clinical data

**DOI:** 10.1002/alz.092688

**Published:** 2025-01-09

**Authors:** Yueqi Ren, Babak Shahbaba, Craig EL Stark

**Affiliations:** ^1^ Mathematical, Computational, and Systems Biology, University of California, Irvine, Irvine, CA USA; ^2^ Medical Scientist Training Program, School of Medicine, University of California, Irvine, Irvine, CA USA; ^3^ Department of Statistics, University of California, Irvine, Irvine, CA USA; ^4^ Department of Neurobiology and Behavior, University of California, Irvine, Irvine, CA USA

## Abstract

**Background:**

Faced with a rapidly aging population and the rising prevalence of Alzheimer’s disease (AD) and related dementias, the field needs to urgently consider screening tools that utilize widely accessible data modalities. We have previously shown that lower‐cost data, operationalized as data modalities accessible at primary care visits, can indeed accurately predict AD clinical diagnosis and that clustering these data can provide useful information. Here, we apply a similar approach to predicting histopathological status.

**Method:**

We first applied our previously‐developed feature extraction method based on a supervised encoder (SE) to transform potentially noisy input features while maintaining or amplifying relevant information. We next performed classification and clustering to stratify subjects by their neuropathology. Here, we compared two traditional classification methods with a novel Bayesian clustering‐classification algorithm called an Infinite Mixture Classifier (IMC). We identified distinct trajectories of subjects based upon changes in cluster assignment over time. Data for this study come from the National Alzheimer’s Coordinating Center, funded by NIA/NIH Grant U24 AG072122 and contributed to by NIA‐funded ADRCs.

**Result:**

We found that relatively high classification accuracy of neuropathologic lesions was possible using widely accessible, lower cost clinical data. In addition, the supervised clusters, derived from using the SE’s latent features and from the IMC, held meaningful clinical diagnostic information that differentiates subjects along the clinical and pathologic continuum. When clusters were derived using longitudinal clinical data, we further observed distinct trajectories of subjects across time as their cluster assignments changed. These trajectory subgroups have significantly different risk of showcasing each type of neuropathologic lesion obtained from postmortem neuropathology.

**Conclusion:**

Our framework benefits from the combined strengths of clustering and classification methods while avoiding drawbacks of unsupervised methods. By using lower cost features, which could be obtained at Medicare annual wellness visits, this work is broadly generalizable and has direct implications for screening of neuropathologic lesions of AD and related dementias for the public. As blood biomarkers become more accessible, our framework can be easily extended to include additional data to improve screening for neuropathology using widely accessible data.

